# Plant Growth Promoting Bacteria Associated with *Langsdorffia hypogaea*-Rhizosphere-Host Biological Interface: A Neglected Model of Bacterial Prospection

**DOI:** 10.3389/fmicb.2017.00172

**Published:** 2017-02-10

**Authors:** Érica B. Felestrino, Iara F. Santiago, Luana da Silva Freitas, Luiz H. Rosa, Sérvio P. Ribeiro, Leandro M. Moreira

**Affiliations:** ^1^Núcleo de Pesquisas em Ciências Biológicas, Universidade Federal de Ouro PretoOuro Preto, Brazil; ^2^Laboratório de Genômica e Interação Microrganismos-Ambiente, Departamento de Ciências Biológicas, Instituto de Ciências Exatas e Biológicas, Universidade Federal de Ouro Preto, Campus Morro do CruzeiroOuro Preto, Brazil; ^3^Laboratório de Ecologia e Biotecnologia de Leveduras, Departamento de Microbiologia, Instituto de Ciências Biológicas, Universidade Federal de Minas GeraisBelo Horizonte, Brazil; ^4^Programa de Pós-Graduação em Biomas Tropicais, Departamento de Biodiversidade, Evolução e Meio Ambiente, Instituto de Ciências Exatas e Biológicas, Universidade Federal de Ouro PretoOuro Preto, Brazil

**Keywords:** *Langsdorffia hypongaea*, bioprospecting, biotechnological potential, plant growth-promoting bacteria, Brazilian Iron Quadrangle, IAA and siderophores

## Abstract

Soil is a habitat where plant roots and microorganisms interact. In the region of the Brazilian Iron Quadrangle (IQ), studies involving the interaction between microbiota and plants have been neglected. Even more neglected are the studies involving the holoparasite plant *Langsdorffia hypogaea* Mart. (Balanophoraceae). The geomorphological peculiarities of IQ soil, rich in iron ore, as well as the model of interaction between *L. hypogaea*, its hosts and the soil provide a unique niche that acts as selective pressure to the evolution of plant growth-promoting bacteria (PGPB). The aim of this study was to prospect the bacterial microbiota of holoparasitic plant *L. hypogaea*, its plant host and corresponding rhizosphere of IQ soil, and to analyze the potential of these isolates as PGPB. We obtained samples of 11 individuals of *L. hypogaea* containing fragments of host and rhizosphere remnants, resulting in 81 isolates associated with Firmicutes and Proteobacteria phyla. The ability to produce siderophores, hydrocyanic acid (HCN), indole-3-acetic acid (IAA), nitrogen (N_2_) fixation, hydrolytic enzymes secretion and inhibition of enteropathogens, and phytopathogens were evaluated. Of the total isolates, 62, 86, and 93% produced, respectively, siderophores, IAA, and were able to fix N_2_. In addition, 27 and 20% of isolates inhibited the growth of enteropathogens and phytopathogens, respectively, and 58% were able to produce at least one hydrolytic activity investigated. The high number of isolates that produce siderophores and indole-3-acetic acid suggests that this microbiota may be important for adaptation of plants to IQ. The results demonstrate for the first time the biological importance of Brazilian IQ species as reservoirs of specific microbiotas that might be used as PGPB on agricultural land or antropized soils that needs to be reforested.

## Introduction

Throughout evolution, plants have developed adaptive mechanisms related to interactions with microorganisms (Zilber-Rosenberg and Rosenberg, 2008). Accordingly, plants comprise a complex host system, made up of different microhabitats that can be simultaneously colonized by a great diversity of endophytic and epiphytic microorganisms (Lodewyckx et al., 2002). This microbial community is essential for the development of plants since it facilitates the absortion of nutrients and at the same time provides protection against phytopathogens (Fungi, oomycetes, bacteria, viroses, protozoa, and nematodes) and herbivores (Lynch and Whipps, 1990).

The rhizosphere or portion of soil that has close contact with the plant roots represents a highly dynamic environment that enables the interaction of roots with beneficial and pathogenic microorganisms, invertebrates and even root systems of other plants (Bais et al., 2006; Raaijmakers et al., 2009). The communication between the plant roots and organisms present in the rhizosphere is based on the production and secretion of chemicals that can cause different responses depending on the sensitivity or responsiveness of microorganisms present in this highly dynamic environment (Jones et al., 1994; Bertin et al., 2003; Badri et al., 2009).

Some microorganisms present in this plant rhizosphere-interface have the ability to solubilize mineral phosphates, among other soil nutrients (Rodriguez and Fraga, 1999). Many of them synthesize, provide or increase the production of plant hormones such as indole-3-acetic acid (IAA), gibberellic acid, cytokines and ethylene (Costacurta and Vanderleyden, 1995); promote associative nitrogen fixation (Richardson et al., 2009); and produce siderophores (Kloepper et al., 1980), hydrolytic enzymes such as glucanases, chitinases, proteases, cellulases, and amylases (Bashan and de-Bashan, 2005), hydrocyanic acid (HCN) (Voisard et al., 1989), and even antimicrobial agents (Compant et al., 2005). All these features allow classify them as plant growth-promoting bacteria (PGPB) (Bashan and Holguin, 1998). Accordingly, plant growth is favored by the influence of the direct or indirect action of these microorganisms, which features them as important biotool of agronomic and environmental interest (Mirza et al., 2001; Ramamoorthy et al., 2001; Vessey, 2003). Besides this potential (Moore et al., 2003), these microorganisms are commercially important when capable of producing enzymes with different applicabilities in specific sectors. In the same way, secondary metabolites produced by these microorganisms have been used in medicine, when they have antibiotic, antitumor, antifungal, or antiparasitic activity (Bertin et al., 2003; Glick, 2010).

Therefore, understanding distinct interactions of the microbiota with soil and plants allow not only a better understanding of the biological models studied, but also prospecting potential uses of this microbiota or even its biomolecules in a biotechnological perspective. In this context, the search for new microorganisms and natural processes in environments with unique characteristics and that are neglected in biological studies are fundamental, and this is the case of the Brazilian Iron Quadrangle. The geomorphological peculiarities of this soil rich in iron ore as well as the model of interaction with plants provide a unique niche that acts as a selective pressure to the evolution of PGPR. Furthermore, these peculiarities make this environment a potential hostspot of microbial diversity. Belonging to a geologically very old craton that covers about 7200 km^2^, the IQ extends between southeast of Ouro Preto and northeast of Belo Horizonte, continuing to the south of Serra do Espinhaço. In this region, there are rocky outcrops that have a naturally high contamination of soil with heavy metals, which makes the environment very adverse for many plant species. Despite this adverse condition, the IQ presents a great floristic diversity with high levels of endemism (Jacobi and do Carmo, 2008). Due to its association with an extensive deposit of iron ore, and since it is one of the least studied ecosystems in Brazil, IQ has a seriously threatened biodiversity due to the intense mining activity associated with its iron outcrops.

**FIGURE 1:**
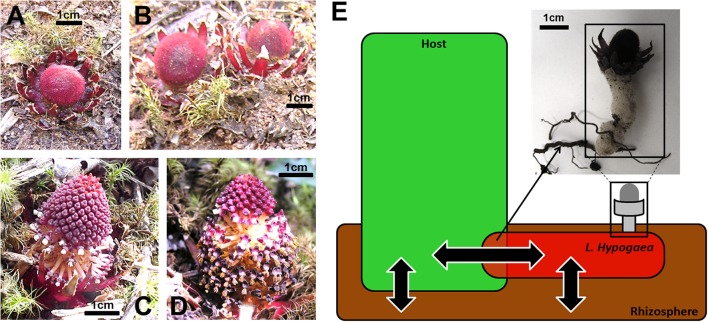
**General features of *Langsdorffia hypogaea.*** **(A)** and **(B)** Feminine inflorescences of *L. hypogaea;* **(C)** and **(D)** Masculine inflorescences of *L. hypogaea*; **(E)** Profile of interactions between *L. hypogaea* and its host, and both interacting with the rhizosphere. As thick arrows indicate the possible flows of substances and microorganisms between niches. The figure image in upper panel displays these physical interactions between *L. hypogaea* and roots of host plant.

Among the plant species threatened by this anthropic activity in IQ, there have been few studies particularly on holoparasitic plants, as in the case of *Langsdorffia hypogaea* MART, the model of this study, being Asteraceae (*Guatteria* genus), Fabaceae (*Dalbergia* genus), Melastomataceae (*Miconia* genus), and Myrsinaceae (*Myrsine* genus) the most representative families of potential host plants from *L. hypogaea* (Vale, 2013). There are approximately 4200 species of parasitic plants distributed in 18 families and 274 genera (Nickrent, 2002). *Langsdorffia hypogaea* is one of the 44 species of plants described belonging to the family Balanophoraceae, which includes herbaceous angiosperms, achlorophyllous plants and holoparasites of roots of trees, shrubs, and even herbaceous plants (Hsiao et al., 1995). In Brazil, this holoparasite is found in the Amazon, Caatinga, Cerrado, and Atlantic Forest (Cardoso, 2014), and although it is not threatened by extinction, due to its wide distribution, it is considered at risk because of substantial habitat loss, due to global warming (Miles et al., 2004) and human use for obtaining wax (Pott et al., 2004). In some places, however, it is considered “Rare,” and it is on the list of threatened flora of the state of Paraná, Brazil (Sema/Gtz, 1995). In fact, a series of local compromising extinctions can be disrupting the gene flow of this species so vagile in its biology of dispersion, and historical events may no longer correspond to the effective state of extinction threat. Morphologically, *L. hypogaea* has two regions that are well distinguishable anatomically, i.e., a basal vegetative body and an apical reproductive region. The vegetative body or rhizome is irregularly cylindrical, elongate and epigeal, with tomentose and fleshy appearance (Hsiao et al., 1995). The reproductive region is represented by dioecious, fleshy, unisexual inflorescences that erupt from ascending vegetative branches, encircle at the base by a sheath of bracts where the fruits are drupaceous and small (Hansen, 1980; Cardoso et al., 2011) (**Figures 1A–D**). Haustoria extend from the vegetative body and attach to the roots of host plants (Nickrent, 2002). Although there is direct connection with its host, part of vegetative body stays surrounded by soil, allowing intimate contact with organisms that live in the rhizosphere (**Figure 1E**).

Taking into account this anatomic-physiological perspective and composition of fixation substrate of the plant described above, some questions involving this model of interaction are yet to be answered to better comprehend the biology of the species: understand if the microbiota living in association with *L. hypogaea* is shared to the host plant as well as with the rhizosphere, and verify if the potential of these bacterial isolates contribute to the adaptive mechanism of these plants in such a hostile environment as the soil from ferruginous fields.

In an attempt to answer these and other related questions, we carried out bacterial prospecting. The main objective was to identify which bacterial species were associated with *L. hypogaea*, the host and specific rhizosphere of the soil of the semidual seasonal forests of Brigida hill, basically composed by sandy-clay textures, low concentration of P and K, high concentration of Ca, Mg, Fe, As, and Sb (Vale, 2013), and pH values ranging from 3.9 to 6.2 in the first 20 cm deep (Filho et al., 2010). In parallel, biochemical assays were used to investigate the biotechnological potential of these isolates, which could be eventually used for various purposes.

## Materials and Methods

### Location and Sampling of Plants and Rhizosphere Soil

The collections were made in Serra da Brigida (central point: 20°21′35′′S, 43°30′11′′W), which is part of Parque Natural Municipal das Andorinhas and is in southern part of Environmental Protection Area Cachoeira das Andorinhas, within IQ (Ferreira, 2011), municipality of Ouro Preto, Minas Gerais – Brazil (**Supplementary Figure S1**). The topography of the region is sustained by itabirites and quartzites. Itabirites are iron formations, metamorphic and strongly oxidized, showing discontinuous bodies with high ore content (>64% Fe) (Rosière and Chemale, 2000). Sandy and flooded soils are absent and they have large amounts of humic substances (Jacobi and do Carmo, 2008). In this forest fragment, we randomly collected 11 individuals of *L. hypongaea*, containing fragments of parasitized roots and remnants of corresponding rhizosphere. The samples were stored in sterile plastic bags and processed on the same day of collection.

### Isolation of Bacteria, Media and Culture Conditions

The samples of *L. hypogaea* were externally disinfected using chlorine solution 2.5% by 2 min. We used for each sample five inner fragments of *L. hypogaea* root (∼1.0 × 0.5 cm) inoculated in Luria-Bertani (LB) medium (Maniatis et al., 1982) containing 0.03 mg/L thiophanate, with pH adjusted to 6.0. The host root was washed following a standardized sequence of solutions for surface disinfection (9 g/L NaCl – 2 min, 70% alcohol – 2 min, 2,5% sodium hypochlorite – 2 min and 9 g/l NaCl – 2 min), and similarly, five fragments of each root of plant host were inoculated in LB medium. For the isolation of microorganisms present in the rhizosphere and in fragments of plant host root containing traces of soil (approximately 2 g), the samples were placed in a 10 mL of saline solution (0,5 NaCl g/L) for 10 min. Next, an aliquot of 100 μL of this solution was inoculated in selective LB medium. All plates were incubated at 25–28°C for a period of up to 10 days, and the microorganisms grown were isolated in new 60 mm diameter Petri dishes containing the same culture medium. The colonies isolated were photographed (front and back, data not shown) and grouped according to their origin. All isolates were cataloged and preserved in 30% glycerol and stored at -80°C.

### DNA Extraction, Amplification, and Sequencing

DNA of the isolates was extracted using the CTAB/NaCl protocol (Doyle and Doyle, 1987). The primers 27f and 1492r were used for amplification of bacterial 16S rRNA gene (Lane et al., 1985). The 50-μL PCR mixture contained 20–50 ng of DNA, 250 pmol of each primer, 5 μL 10x PCR buffer, 2.5 U rTaq DNA polymerase^TM^ (Invitrogen), and 100 μM deoxynucleoside triphosphate mixture. The PCR program consisted of initial denaturation at 95°C for 5 min, followed by 35 cycles of 1 min of denaturation at 95°C, 45 s of annealing at 47°C and 2 min of extension at 72°C and a final extension for 10 min at 72°C, utilizing a 2720 ThermalCycler^TM^ (Applied Biosystems). The amplicons generated by PCR were verified in 1% agarose gels and purified using 20% PEG-8000 in 2.5 M NaCl (Arbeli and Fuentes, 2007). The product obtained was quantified by spectrophotometry using a NanoDrop ND 1000^TM^ (NanoDrop Technologies). Sequencing was carried with the DYEnamicTM^TM^ kit (Amersham Biosciences, USA) in combination with the MegaBACE 1000^TM^ automated sequencing system (Amersham Biosciences, USA). The sequencing reactions were performed with 100–150 ng purified DNA and the reagents in the DYEnamicTM^TM^ kit (Amersham Biosciences, USA), using the manufacturer’s recommendations. The program consisted of 36 cycles with an initial denaturation at 95°C for 25 min, followed by 15 s of annealing at 50°C and 3 min of extension at 60°C. After cycling, the reaction product was transferred to a 96-well sequencing plate to be precipitated.

For precipitation, 1 μL of 7.5 M ammonium acetate was added to each well. Next, 28 μL of absolute ethanol (Merck, USA) were added. The plate was vortexed and incubated for 20 min at room temperature, protected from light. Afterward, the plate was centrifuged for 45 min at 3200 × *g* and the supernatant was discarded. Next, 150 μL of 70% ethanol were added. The plate was centrifuged again for 15 min at 3200 × *g* and the supernatant was then discarded. The plate was allowed to stand for 20 min, protected from light, para evaporation of ethanol. Precipitated DNA in each well of the plate was then resuspended in 10 μL of loading buffer (present in sequencing kit). The plate was vortexed for 2 min, centrifuged for 1 s at 800 × *g* and stored at 4°C, protected from light, until injection of samples in a MegaBACE 1000^TM^ sequencer (Amersham Biosciences, USA).

### Determination of Sequences and Phylogenetic Analysis

The contigs were assembled using the forward and reverse sequences of each 16S rRNA gene amplicon using Phred (Ewing and Green, 1998; Ewing et al., 1998). The DNA sequences were analyzed utilizing the BLASTn program (Altschul et al., 1997). The sequences were aligned using the program Muscle (Edgar, 2004) and then curated by the program G-block (Castresana, 2000). A neighbor-joining phylogenetic tree was then determined using PhyML 3.0 (Anisimova and Gascuel, 2006) followed by TreeDyn (Chevenet et al., 2006), and the statistical robustness of the analysis was estimated by bootstrapping with 1,000 replicates.

### Nucleotide Sequence Accession Numbers

All sequences obtained in this study were deposited in GenBank, according to the accession numbers given in **Table 2**.

### Production of IAA

The production of IAA was determined by the method of Bric et al. (1991), with modifications. Bacterial producers of IAA were identified by the change in the color of the nitrocelulose disk from yellow (negative result) to red (positive result). The assays were made in triplicates and only those that achieved a positive result in at least two of them were accounted in the analysis.

### Production of Ammonium Ions

The production of ammonium ions was determined by the indophenol method (Verdouw et al., 1978), using *Proteus* sp. as positive control (Vince et al., 1973). The samples were read at 690 nm, and the absorbance values were compared with a control condition. The assays were made in triplicates and only those that the absorbance values were greater than or equal to the *Proteus* values in at least two of the assays were accounted in the analysis.

### Production of Siderophores

The production of siderophores was based on the work of Schwyn and Neilands (1987), with modifications. To remove traces of iron in medium it was made a pretreatment with hidroxiquinolone followed by separation using dropping funnel (Pierre et al., 2003). An orange shade of the culture medium around the regions where bacteria grew was indicative of production of siderophores. The assays were made in triplicates and only those that achieved a positive result in at least two of them were accounted in the analysis.

### Nitrogen Fixation

Nitrogen fixation capacity was investigated using two serial methods: Nitrogen-free combined carbon (NFCC) semi-solid medium, free of N_2_ (pH 5.7), supplemented with 5 g/L mannitol and 5 g/L sucrose (Dobereiner et al., 1976) and PCR using universal *nifH* primers (Burgmann et al., 2004). The Change in color of medium from yellow to green was indicative of the capacity to fix N_2_. This assay was made in triplicates and only those that achieved a positive result in at least two of them were accounted in the analysis, which was confirmed by amplification of *nifH* fragment evaluated in 1.2% agarose gel. *Bradyrhizobium elkanii* BR96 was used as positive control.

### Production of Hydrocyanic Acid (HCN)

The production of HCN was determined by the method of Bakker and Schippers (1987), with modifications. A change in yellow color of filter paper (negative result) to brown (positive result) indicated production of HCN. The assays were made in triplicates and only those that achieved a positive result in at least two of themwere accounted in the analysis.

### Production of Amylase, Cellulase, and Protease

Amylase activity was determined in 90-mm Petri dishes containing Yeast nitrogen base (YNB) medium (2%) containing 2 g/L soluble amide, 5 g/L peptone, and 1 g/L yeast extract, with pH adjusted to 6.0 (Strauss et al., 2001). Cellulase activity was determined in 90-mm Petri containing YNB medium (2%) supplemented with 0.5 g/L cellobiose and 1 g/L carboxymethyl cellulose (Teather and Wood, 1982). Protease activity was determined in 90-mm Petri dishes containing culture medium composed of 20 g/L casein, 5 g/L peptone, 3 g/L yeast extract, 10 g/L glucose, and 20 g/L agar, with pH adjusted to 5.0 (Strauss et al., 2001). After 3 days of growth at 28°C, the bacterial isolates in all assays producing a transparent halos determined the amylase, cellulose, or protease activity in the respective isolates and assays.

### Inhibition of Entero- and Phytopathogens

To investigate a possible antimicrobial activity of the isolates, antimicrobial assays were performed against the following targets: *Staphylococcus aureus* ATCC 29213 (opportunistic human pathogen), *Bacillus cereus* ATCC 11778 (associated with food poisoning), *Klebsiella pneumoniae* ATTC 4352 (causal agent of pneumonia), *Shigella flexneri* (associated with dysentery) and *Xanthomonas citri* subsp. *citri* 306 (causal agent of citrus canker) using solid LB in a direct inhibition test. The assays were made in triplicates and only those that achieved a positive result in at least two of them were accounted in the analysis.

To investigate the capacity of isolates to inhibit the growth of the phytopathogenic fungus *Fusarium oxysporum* f.sp. *lini* (causal agent of various diseases in plants), the isolates were grown in LB and then transferred to 60-mm Petri dish containing potato agar. The bacterial isolates were inoculated to make a square of approximately 2 cm on the culture medium. After 2 days of growth of these isolates at 28°C, a fraction of 4 mm^2^ of culture medium containing *F. oxysporum* was inoculated exactly at the center of the plate (center of square), starting at a pre-growth of 5 days at 28°C. The bacteria capable of producing some anti-*Fusarium* substance hindered the growth of the fungus, compared to the growth profile of *Fusarium* under control conditions in the absence of bacterial isolate. The values to determine per cent inhibition were obtained by the formula (%) = (C × E)/C × 100, where *C* is the diameter of the *Fusarium* culture in control, and *E* is the diameter in the presence of bacterial isolate (Zhao et al., 2014). The assays were made in triplicates and the average of per cent inhibition was ploted in radar graph.

## Results

### Characterization of Bacterial Population

Of the 11 samples collected in four different points of Serra da Brigida-MG, we selected and preserved 81 isolates (**Table 1**). However, isolate L5 was non-viable after storage in glycerol. The growth rate of isolates L13, L15, L21, H57, and H79 were unsatisfactory, resulting in the extraction of insufficient DNA and sequencing of low quality. Although sample L4, isolated from *L. hypogaea*, showed good quality of sequencing and satisfactory assembly of contigs using the assembly parameters (see Materials and Methods), it still showed low percentage of similarity (76.8%) and identity (77%) compared with sequences deposited in GenBank of NCBI, featuring it as a potential new organism to be investigated rigorously.

**Table 1:** Distribution of bacterial representativeness of taxa found in *Langsdorffia hypogaea*, host and rhizosphere.

Phylogenetic group	Bacteria	Number of isolates (Shared with other niches)		
		*L. hypogaea*	Host	Rhizosphere
Firmicutes	*Bacillus cereus*	1	2 (2)	3 (2)
	*Bacillus mycoides*	1		
	*Bacillus* sp.	3 (2)	4 (3)	2 (1)
	*Lysinibacillus sphaericus*	4 (2)	1	
	*Lysinibacillus xylanilyticus*	1	1	1
	*Lysinibacillus* sp.		3 (2)	3 (3)
	*Viridibacillus arenosi*			1
	*Paenibacillus taichungensis*		3 (2)	

Gammaproteobacteria	*Citrobacter freundii*		1	
	*Enterobacter aerogenes*	1		
	*Enterobacter* sp.	1	1	2(1)
	*Klebisiella oxytoca*	1		
	*Klebisiella* sp.	1		1
	*Pseudomonas fluorescens*			1
	*Shewanella* sp.		1	
	*Raoultella terrigena*			1
	*Rahnella* sp.	1		
	*Rahnella aquatilis*			1
	*Serratia marcescens*		2 (2)	1
	*Serratia proteamaculans*	3 (2)	5 (2)	8 (2)
	*Serratia* sp.		1	6 (2)

TOTAL	21	18 (6)	25 (13)	31 (11)

In the three niches analyzed, we found isolates of the phyla Firmicutes (34), belonging to the genera *Bacillus* (17), *Lysinibacillus* (13), *Paenibacillus* (3), and *Viridibacillus* (1), and Proteobacteria (40), belonging to the genera *Serratia* (26), *Klebsiella* (3), *Rahnella* (2), *Citrobacter* (1), *Enterobacter* (5), *Shewanella* (1), *Raoultella* (1), and *Pseudomonas* (1) (**Table 2**). Four of these genera (*Serratia, Bacillus, Lysinibacillus*, and *Enterobacter*) were found in all three niches analyzed, where the first genera was the most representative. Isolates of the genera *Citrobacter, Paenibacillus*, and *Shewanella* were found only in the plant host. Isolates of the genera *Viridibacillus, Pseudomonas*, and *Raoultella* were found only in the rhizosphere (**Table 2**). Isolates of the genera *Klebsiella* and *Rahnella* were found exclusively shared between *L. hypogaea* and the rhizosphere.

**Table 2:** List of isolates representing the 75 OTUs from *L. hypogaea*, host and rhizosphere.

Representative isolate (Accesion number)	Nearest type strain	Seq. Id (%)	Q. Cover (%)	Accession number
***L. hypogaea***				
L6 (KU057004)	*Bacillus mycoides* UrCA07	73	89	KC618478
L7 (KU057005)	*Bacillus* sp. SG19	96	99	JX402434
L18 (KU057014)	*Bacillus* sp. SG19	96	99	JX402434
L19 (KU057015)	*Bacillus sp. WYT007*	95	98	JQ807855
L22 (KU057017)	*Bacillus cereus HKG201*	69	85	KF947110
L8 (KU057006)	*Lysinibacillus sphaericus* R7	95	98	HQ259956
L9 (KU057007)	*Lysinibacillus sphaericus* R7	96	95	HQ259956
L10 (KU057008)	*Lysinibacillus sphaericus* R7	94	94	HQ259956
L14 (KU057011)	*Lysinibacillus sphaericus* DS11	96	99	EU835735
L20 (KU057016)	*Lysinibacillus xylanilyticus* TAX5	95	99	JX280924
L11 (KU057009)	*Enterobacter aerogenes* PSB28	95	99	FJ360760
L17 (KU057013)	*Enterobacter* sp. SPj	95	93	FJ405369
L16 (KU057012)	*Klebsiella oxytoca ALK313*	97	98	KC456529
L28 (KU057023)	*Klebsiella* sp. 38	96	90	EU294412
L12 (KU057010)	*Rahnella* sp. DmB	95	97	KF720908
L1 (KU057001)	*Serratia proteamaculans* 568	97	99	NR074820
L2 (KU057002)	*Serratia proteamaculans* PW172	94	98	JF494823
L3 (KU057003)	*Serratia proteamaculans* PW172	93	98	JF494823
L4	No significant similarity found.	—–	—–	
**Host**				
H61 (KU057055)	*Bacillus cereus* M2	95	99	JF836882
H65 (KU057059)	*Bacillus* sp. SG19	96	94	JX402434
H69 (KU057063)	*Bacillus* sp. SG19	97	99	JX402434
H72 (KU057066)	*Bacillus* sp. S11714	99	99	KF956655
H77 (KU057071)	*Bacillus cereus strain D7*	97	99	KF500919
H80 (KU057073)	*Bacillus* sp. N4/130	95	98	LN680100
H56 (KU057051)	*Lysinibacillus* sp. NSi08	42	79	AB811363
H73 (KU057067)	*Lysinibacillus* sp. E15	72	94	JN082735
H63 (KU057057)	*Lysinibacillus* sp. E15	95	94	JN082735
H78 (KU057072)	*Lysinibacillus xylanilyticus* fwzy21	95	99	KF208475
H81 (KU057074)	*Lysinibacillus sphaericus* STNG28	95	92	KF312283
H59 (KU057053)	*Paenibacillus taichungensis* B2	95	99	JX010966
H66 (KU057060)	*Paenibacillus taichungensis* B2	96	98	JX010966
H70 (KU057064)	*Paenibacillus taichungensis* A80	86	79	JX010963
H64 (KU057058)	*Citrobacter freundii* H1-2	71	95	KC210870
H60 (KU057054)	*Shewanella* sp. *XH15*	81	87	KJ922531
H58 (KU057052)	*Enterobacter* sp. *RA-15*	97	95	KJ152098
H55 (KU057050)	*Serratia proteamaculans* KB22	96	94	JF327454
H62 (KU057056)	*Serratia proteamaculans* 568	95	99	NR_074820
H67 (KU057061)	*Serratia proteamaculans* KB22	95	99	JF327454
H68 (KU057062)	*Serratia proteamaculans* 568	94	93	NR074820
H71 (KU057065)	*Serratia proteamaculans* 568	96	99	NR074820
H74 (KU057068)	*Serratia marcescens* S418	96	99	GQ202220
H75 (KU057069)	*Serratia marcescens* subsp. *sakuensis* RK26	96	99	KC790279
H76 (KU057070)	*Serratia marcescens* subsp. *sakuensis* RK26	95	90	KC790279
**Rhizosphere**				
R24 (KU057019)	*Bacillus* sp. SG19	95	99	JX402434
R31 (KU057026)	*Bacillus cereus* M2	95	99	JF836882
R32 (KU057027)	*Bacillus cereus* S72	96	99	FJ763650
R35 (KU057030)	*Bacillus cereus* S*72*	96	99	FJ763650
R49 (KU057044)	*Bacillus* sp. SG19	95	99	JX402434
R40 (KU057035)	*Viridibacillus arenosi* Hc6	95	99	JF899298
R23 (KU057018)	*Lysinibacillus* sp. O-E16	95	99	JN613478
R29 (KU057024)	*Lysinibacillus xylanilyticus* fwzy21	71	87	KF208475
R33 (KU057028)	*Lysinibacillus* sp. T1-9	95	89	KJ127177
R34 (KU057029)	*Lysinibacillus* sp. E15	97	89	JN082735
R39 (KU057034)	*Klebsiella* sp. D81	96	97	DQ923489
R48 (KU057043)	*Enterobacter* sp. Hg4-01	94	97	EU304247
R38 (KU057033)	*Enterobacter* sp. Hg4-01	83	96	EU304247
R37 (KU057032)	*Raoultella terrigena strain PK35*	97	97	KC790281
R47 (KU057042)	*Rahnella aquatilis* 2B-CDF	95	99	FJ811859
R25 (KU057020)	*Serratia* sp. PT3	95	99	GU458285
R26 (KU057021)	*Serratia* sp. PT3	95	99	GU458285.2
R27 (KU057022)	*Serratia* sp. PT3	94	98	GU458285.3
R30 (KU057025)	*Serratia* sp. S3.MAC.008	70	90	HM063908
R36 (KU057031)	*Serratia proteamaculans* KB22	95	99	JF327454
R41 (KU057036)	*Serratia marcescens* KtMC2-16	97	99	KC122200
R42 (KU057037)	*Serratia* sp. PT3	95	99	GU458285
R43 (KU057038)	*Serratia proteamaculans* KB22	94	99	JF327454
R44 (KU057039)	*Serratia proteamaculans* 568	94	96	NR074820
R45 (KU057040)	*Serratia* sp. PT3	96	96	GU458285
R46 (KU057041)	*Serratia proteamaculans* KB22	96	98	JF327454
R50 (KU057045)	*Serratia proteamaculans* KB22	94	99	JF327454
R51 (KU057046)	*Serratia proteamaculans* 568	95	98	NR074820
R52 (KU057047)	*Serratia proteamaculans* 568	95	98	NR074820
R53 (KU057048)	*Serratia proteamaculans* 568	95	96	NR074820
R54 (KU057049)	*Pseudomonas fluorescens* Y5	74	97	KJ882377

**FIGURE 2:**
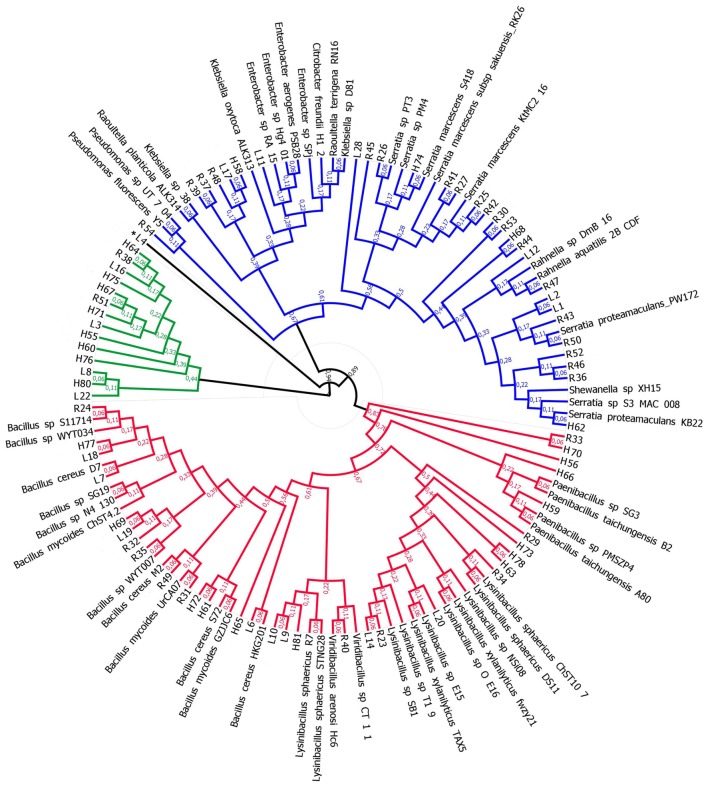
**Phylogenetic analysis of isolates**. Two large clades were identified, Proteobacteria (blue) and Firmicutes (red). An isolated clade was established including some isolates obtained in this study. ^∗^Denotes the sequences that did not form a contig, using the pre-established assembly parameters (see Materials and Methods).

Phylogenetic analysis of the isolates confirmed the sequencing results and allowed the grouping of the bacterial isolates from different niches to the taxa represented, Firmicutes and Proteobacteria. The isolates from *L. hypogaea* (L3, L16, L22, and L8), the isolates from the plant host (H55, H60, H64, H67, H71, H75, H76, and H80), and the isolates from the rhizosphere (R38 and R51) were grouped in a different clade (**Figure 2**).

**FIGURE 3:**
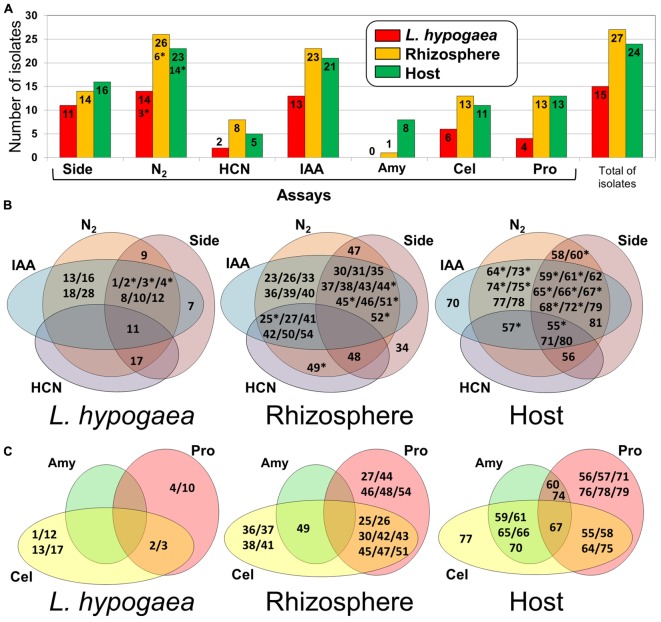
**Analysis of biochemical and enzymatic assays. (A)** Representativeness of isolates with regard to results of biochemical assays for production of siderophores (Side), N_2_ fixation (N2), production of HCN (HCN), production of IAA (IAA), and positive activity for amylase (Amy), cellulase (Cel), and protease (Pro). **(B)** Venn diagrams indicating the potential of each isolate with regard to the biochemical performed (IAA, HCN, N2, and Side) in each niche evaluated (Langs, Rizhos, and Host). **(C)** Venn diagrams showing the potents of each isolate with regard to the enzymatic assays performed (Cel, Pro, and Amy) in each niche evaluated (Langs, Rhizos, and Host). ^∗^Denotes the isolates whose *nifH* were amplified by PCR.

For the biochemical tests, 66 isolates were analyzed since nine of these isolates showed reduced growth rate, prevented an accurate analysis of the results in the proposed biochemical assays. Of these, 63 isolates showed growth in nitrogen-free medium, representing 14 (93.4%) of the 15 isolates of *L. hypogaea*, 23 (95.8%) of the 24 isolates from plant host and 26 (96.3%) of the 27 isolates from rhizosphere (**Figure 3A**). From these isolates, the presence of *nifH* was confirmed in 23 genomes by PCR analysis (**Supplementary Figure S2**). With regard to siderophore production, more than 62% of the isolates were capable of producing these compounds. All isolates were able to produce ammonium ions, at different concentrations. Meanwhile, only 15 isolates (22.72%) were capable of producing hydrocyanic acid. More than 86% of the isolates were capable of producing IAA, of which 13 were from *L. hypogaea* (**Figure 3A**). In attempt to understand the potential of each of these isolates with regard to the capacity to fix atmospheric N_2_, to produce siderophores, IAA, and HCN, Venn diagrams were prepared for each of the three niches analyzed (**Figure 3B**). Especially the isolates L11, H55, H71, and H80 yielded positive results for all analyses. Of the isolates obtained from *L. hypogaea*, four were capable of to fix N_2_ and to produce IAA (L13, L16, L18, and L28), and seven were able to produce IAA and siderophores, and to fix N_2_ (L1, L2, L3, L4, L8, L10, and L12). Of the isolates obtained from the rhizosphere, six were capable of producing IAA and fixing N_2_ (R23, R26, R33, R36, R39, and R40). Eleven were capable of producing IAA and siderophores, and even fixing N_2_ (R30, R31, R35, R37, R38, R43, R44, R45, R46, R51, and R52), while another six were capable of producing IAA and HCN, and fixing N_2_ (R25, R27, R41, R42, R50, and R54). Of the isolates obtained from the host plant, six were capable of producing IAA and fixing N_2_ (H64, H73, H74, H75, H77, and H78). Two were capable of producing siderophores and fixing N_2_ (H58 and H60), whereas ten were able to produce IAA and siderophores, and to fix N_2_ (H59, H61, H62, H65, H66, H67, H68, H72, H79, and H80).

Regarding hydrolytic enzymes assay, 11 isolates were able to produce amylase. None of these isolates was obtained from *L. hypogaea*, the isolates obtained from the plant host were the most representative in this analysis. Thirty isolates were capable of producing cellulase, with greatest representativeness among the isolates from the rhizosphere. Thirty isolates were able to produce proteases, with egual representativeness among the isolates from the plant host and rhizosphere. Similarly, a Venn diagram pointing out the isolates and their origin with respect to the production of hydrolytic enzymes was constructed (**Figure 3C**). Notably, isolate H67 was capable of producing all three types of hydrolytic enzymes investigated.

**FIGURE 4:**
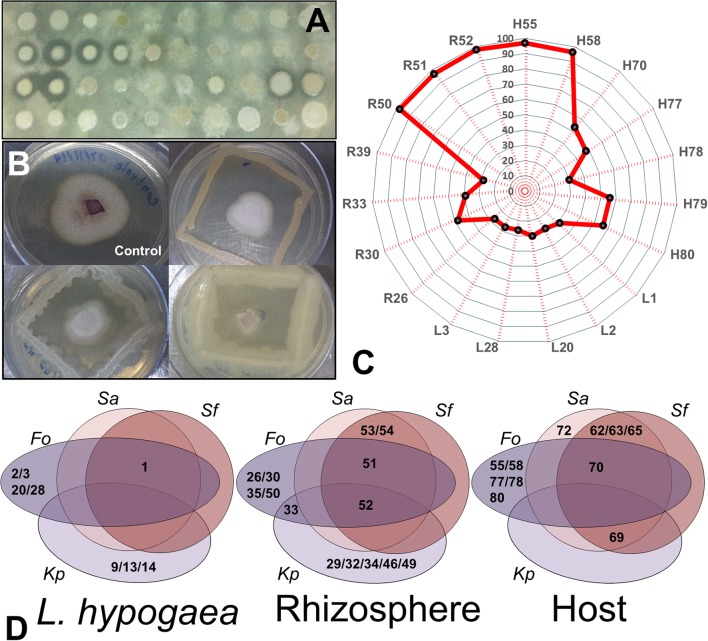
**Analysis of inhibition of entero- and phytopathogens. (A)** Profile of growth inhibition of isolates regarding the target *S. aureus*. **(B)** Profile of growth inhibition of *Fusarium* induced by three of the 19 isolates positive for this analysis. **(C)** Radar demonstrating the representativeness and percentage of growth inhibition of *Fusarium* of the 19 isolates positive for this analysis. Note that the majority of isolates from the rhizosphere inhibited almost 90% of growth of the fungus. **(D)** Venn diagrams showing the potentials of each isolate with regard to inhibition of enteropathogens (Sa, *S. auereus*; Sf, *S. flexneri;* and Kp, *K. pneumoniae*) and of *Fusarium oxysporum* (Fo) in each niche evaluated (Langs, Rizhos, and Host).

Twenty isolates were able to inhibit the growth of enteropathogens. They include 11 inhibitors of *Staphylococus aureus* (L1, R51, R52, R53, R54, H62, H63, H65, H69, H70, and H72), 11 inhibitors of *Klebisiella pneumoniae* (L9, L13, L14, R29, R32, R34, R46, R49, and H69), and 10 inhibitors of *Shiguella flexneri* (L1, R51, R52, R53, R54, H62, H63, H65, H69, and H70) (**Figure 4A**). No isolate was capable of inhibiting the growth of *Bacillus cereus*, and only one isolate inhibited the growth of *Xanthomonas citri* strain 306 (H65). Nineteen isolates inhibited the growth of *Fusarium* (**Figure 4B**), 5 of themwere obtained from *L. hypogaea* (L1, L2, L3, L20, and L28), eight from the rhizosphere (R26, R30, R33, R39, R50, R51, R52, and R55) and six from the plant host (H58, H70, H77, H78, and H80) (**Figure 4D**). The potential of some of these isolates is indicated in **Figure 4C**, ranging from 28% in isolates from *L. hypogaea* to above 95% in the isolates R50, R51, R52, H55, and H58 from rizhosphere and host, respectively.

## Discussion

### Identification of Microbiota Associated with *L. hypogaea* and Its Interactions

The importance and complexity of the rhizosphere in the interaction with plants and other organisms in which they live have been reported in many studies (Bais et al., 2006; Vacheron et al., 2013; Zhang et al., 2014). Similarly, the modifications in the physiological profile of plants as a result of alterations in the chemical composition of the rhizosphere or the microbiota contained therein have also been characterized, especially with regard to the interaction between plants of agricultural interest (Saleem et al., 2007; Bhattacharyya and Jha, 2012; Dodd and Ruiz-Lozano, 2012; Nadeem et al., 2014). Contrary to these advances, studies seeking to understand the identification of microbiota associated with parasitic plants, especially holoparasitic ones are limited or lacking. This relation of strict dependence on its host makes it a very interesting target for understanding the degree of importance of microbiota associated with the adaptation and resultant survival of species in the most diverse environments where they occur. Accordingly, this is the first work aimed at understanding the composition and importance of microbiota in the biological interface holoparasitic plant-host-rhizosphere, using *L. hypogaea* as a model. Not only is the biological model distinctive for studies bioprospecting, the sampling site, the IQ, is characterized as an environment also neglected in studies involving its microbiota. Because it is an environment enriched in iron compounds and other heavy metals, whose soils are extremely compacted, adaptation of this microbiota can be directly related not only to the soil features, but also related to the adaptation of plants in this environment, and may uncover new organisms and the biotechnological potential of this specific microbiota.

Although there are no known genera that are unique for *L. hypongaea*, isolate L4 did not show significant identity (over 80%) with any other isolate with sequence deposited in databanks and therefore deserves attention. This isolate was capable of producing ammonium ions in high concentration, IAA and siderophores and fix nitrogen. This activity could generate better or complementary nutritional conditions compared to those offered by the plant host such as for a tree growing on soils lacking nutrients. A key adaptive aspect of this would be the decrease in risk of mortality of the plant host. Regardless of nutrients that it provides, physical sustentation and supply of water and carbohydrates to the parasite is totally dependent on host plants, and their death is a great adaptive disadvantage (Lopez Pascua et al., 2014). Thus, the co-association with a microbiota capable of providing the necessary nutrients saves the parasitized tree from irreversible stress. However, more studies are necessary to confirm these preliminary results.

Similarly, isolates of the genera *Klebisiella* and *Rahnella* were found only in *L. hypogaea* and the rhizosphere. This allows us to infer that there may be a direct association of soil bacteria with the holoparasitic plant, and that a possible relation of complementarity makes the parasitic plant not only dependent on the plant host for survival but also dependent on specific soil bacteria. This allows us to raise the prospect that perhaps the concept of botanical holoparasitism needs to be rethought, taking the scientific community to undertake further research in this area of knowledge. Although need the host plant for aquisition of carbohydrates, since it does not have the capacity of producing them itself (by the chlorophyll absence), *L. hypogaea* could also depend closely on PGPB to assist in the development of its roots, aquisition of ions from the rhizosphere or even in the interaction with its host plant through their haustoria. Finally, isolates of the genera *Serratia*, *Bacillus*, *Lysinibacillus*, and *Enterobacter* were found in the three niches, reinforcing this exchange of microbiota, now a more complex perspective. These results reinforce the prospect that in a community, the ecological interaction of plants and microorganisms is directly related to the rigor of the habitat and the ability of colonization. In other words, the mutualistic interactions with microorganisms would be the basis of evolution of adaptations to inhospitable environments. The theoretical concept of species “A” of adversity strategists [sensu (Greenslade, 1983)], in contrast to the artificial r-k continuum of Pianka (1970; Taylor et al., 1990), or the understanding of “template habitats” (Southwood, 1977; Korfiatis and Stamou, 1999) and specialization in habitats (consequently, the whole concept and use of bio-indicators) can make sense only in the light of these interactions. Therefore, a specific habitat provides characteristic conditions that promote the growth of certain microorganisms, which in turn facilitate the development of other species associated with them.

Thus, much less time adjustment to selective pressures imposed by oligotrophic and contaminated soils would be necessary to ensure the invasion of environment, always done by species that evolve from less hostile niches. This possibility of having one microbiological micro-habitat that minimizes the natural hostility of the environment can change the ecological-evolutionary perception of biodiversity evolution, and even the basic theoretical models that guide our understanding of these processes.

### PGP Activities of the Isolates

About 95% of isolates showed growth in medium combined nitrogen, thereby demonstrating diazotrophic activity. This may have a direct relation with an environment whose soil is highly leached, oligotrophic and contaminated, like these montane ecosystems or any other soil of Brazilian savannas (Goodland, 1971; Goodland and Pollard, 1973; Batmanian and Haridasan, 1985; Haridasan, 2008). The understanding of the evolutionary costs of colonizing these environments are well studied (Ribeiro and Brown, 2006) and consistent with the existing theoretical propositions (Herms and Mattson, 1992; Fine et al., 2004). This is the first time that the role of mutualistic microbiota was taken into consideraction as a fundamental adaptive mechanism.

Iron can be available in the soil as Fe^2+^ or Fe^3+^, where the latter is less soluble but more abundant. Siderophores are molecules secreted by some bacterial species that chelate Fe^3+^ converting it to Fe^2+^, which as a consequence is internalized by specific cellular receptors for these ions (Neilands, 1995). From a competition point of view, microorganisms that are able to utilize siderophores as a mechanism of acquisition of Fe^3+^ make it available for them consequently decreasing the availability of iron for possible competing microorganisms in the same niche (Hibbing et al., 2010). Often when this competition occurs because of a phytopathogenic organism, this resource becomes used as an indirect mechanism of plant growth since it controls the growth of these phytopathogens (Perez-Montano et al., 2014). In the same perspective, many plants only have receptors for siderophores, depending intimately on the their production by microorganisms that live in symbiosis or cooperation with these plants for acquiring iron from the environment, essential for their growth (Crowley et al., 1991). More than half of the bacterial isolates obtained here were producers of siderophores (**Figure 3**). Although this number was high, it was expected since the environment favors the adaptation of bacteria capable of surviving in such high concentrations of iron. Therefore, further study of the structural composition and regulation of the synthesis of these substances in these microorganisms is necessary and may lead to the discovery of new biomolecules with ion-chelating activity. With regard to the region where the plants were isolated, this perspective becomes even more interesting, because it is an environment classically reported as rich in arsenic, and it is possible that these microorganisms make use of these molecules as an adaptive alternative to the presence of this element (Gonçalves and Lena, 2013).

Similarly, about 86% of isolates were capable of producing IAA. Since this hormone helps to increase root growth, this could explain the adaptation of plants of these niches to extremely hard soil, which provides surface branching of roots, favoring not only anchoring but also procurement of water and minerals. These bacteria may have great importance in the study model proposed since it is a holoparasitic plant whose capacity to produce this hormone can be rather low in relation to an autotrophic plant (Magnus et al., 1982), which could explain its intimate dependence on IAA-producing bacteria.

Far less representative, but of similar importance were the isolates in which we identified hydrolytic enzyme activities. These enzymes are of great industrial interest, since they can optimize the manufacture of products of economic interest such as in the case of glucose for fermentation processes obtained through cellulolytic or amylolytic activity, or of amino acids and peptides widely used in the food, pharmaceutical and chemical industry, obtained from proteolytic activity (Dalmaso et al., 2015). In an environmental microbiological context, all these enzymes can be of fundamental importance in the process of adaptation to a specific niche.

Individually, some genera deserve attention because of the previous results described in the literature. R54, for example, was isolated from the rhizosphere and showed similarity to *Pseudomonas fluorescens.* A recent study involving the strains PA4C2 and PA3G8 of *P. fluorescens*, also isolated from the rhizosphere, were found to be able to inhibit the growth of the phytopathogen *Dickeya* (Cigna et al., 2015), which causes diseases in herbaceous plants. In out study, R54 was also found to be a potential inhibitor of enteropathogens since it blocked the growth of *Staphylococcus aureus* and *Shigella flexneri*. Specific strains of *Pseudomonas* have also been described as inducing systemic resistance in cloves, cucumbers, radishes, tobacco, and *Arabidopsis*, which raisethe possibility of these bacteria being a potential growth inhibitors of phytopathogens in wild plants. Similarly to *Pseudomonas*, induced systemic resistance has also been described for different strains of *Bacillus* spp., including the specific species *B. amyloliquifaciens, B. subtilis, B. pasteurii, B. cereus, B. pumilus, B. mycoides*, and *B. sphaericus* (Choudhary et al., 2007). When inoculated or present in specific organisms, they induce a significant reduction in the incidence or severity of diseases in various hosts (Choudhary et al., 2007). In this study, various isolates showed similarity with bacteria of the genus *Bacillus*, including the strains *B. cereus* and *B. mycoides.* Some of these isolates showed positive results for all the biochemical assays performed (H80) or inhibited the growth of three of the four enteropathogens investigated (H69).

Bacteria of the genus *Enterobacter* has been associated with numerous biological models. *Enterobacter* sp. strain *EJ01* isolated from *Dianthus japonicus thunb* (China Sea rose) was described as a bacterium capable of aiding vegetative growth, besides alleviating salt stress in tomato and *Arabidopsis* (Kim et al., 2014). Of the five isolates similar to *Enterobacter* identified in this study, all produced siderophores and fixed N_2_, and only two were capable of producing IAA. In another study, *Serratia marcescens* isolated from the rhizosphere of the coconut tree was found to fix nitrogen and to produce IAA and siderophores, among other compounds investigated (George et al., 2013), highlighting the importance of these genera in the support and growth of these plants. Of the 26 isolates that showed similarity to *Serratia*, 25 isolates were capable of fixing N_2_ and producing IAA, and 15 were capable of producing siderophores.

*Paenibacillus yonginensis* DCY84 was evaluated in growth with *Arabidopsis thaliana* subjected to salt, drought and heavy metal stress, and the study showed that plants treated with this bacterial isolate were more resistant than the untreated control plants (Sukweenadhi et al., 2015). Our isolates H59, H66, and H70 showed similarity to this genus and were able to produce siderophores and IAA and to fix N_2_, besides inhibiting enteropathogens. Another recent work isolated bacteria from the rhizospheric soil of *Populus euphratica* and identified ten strains that induced a significant increase in dry weight of buds and roots of wheat (Wang et al., 2014). These isolates were identified as being from the genera *Pseudomonas*, *Bacillus*, *Stenotrophomonas*, and *Serratia*. Among these strains, *Serratia* sp. 1–9 and *Pseudomonas* sp. 23/05 were the most effective strains. Both produced auxin, and significantly increased production when grown under simulated dry conditions, leading to a direct effect on promoting plant growth under drought stress (Wang et al., 2014). Similarly, a work identified 12 endophytic bacteria characterized as diazotrophic, two species belonging to the genus *Paenibacillus*, three to the genus *Mycobacterium*, three to the genus *Bacillus*, and four to the genus *Klebsiella* (Ji et al., 2014). Rice seeds treated with these bacteria showed improved growth, increase in height and dry weight and antagonistic effects against pathogenic fungi (Ji et al., 2014). Our isolates that showed high identity to *Paenibacillus* (H59 – 99% and H66 – 98%), were also capable of fixing N_2_ and were thus diazotrophic. The isolate H70, although showing similarity to *Paenibacillus*, was not able to fix N_2_, but did inhibit the growth of enteropathogens and *Fusarium*. All isolates that showed similarity to *Klebisiella* (L16, L28, and R39) were also capable of fixing N_2_, besides producing IAA.

### Perspectives of Use of Isolates

In an agroecological context, there is currently an emerging demand for the development of sustainable agriculture, to decrease our dependence on agrochemical farming and its harmful consequences to the environment (Bhardwaj et al., 2014). The utilization of PGPB to increase farm production has become an important alternative. Similarly, alternative methods for pest control attracted attention, and biological control has been considered a viable solution for various diseases that are difficult to control (Cespedes et al., 2015). This practice aims to maintain a balance in the agroecosystem, so that the host, in the presence of a pathogen or pest, does not suffer significant damage due tothe controlling action exerted by non-pathogenic organisms (Meldau et al., 2012). Thus, understanding microbial relations in soils and plants can lead to the discovery of microorganisms with great agricultural potential and other applications as well.

Besides agroecological importance, all potential presented by microbiota isolated from these neglected biological niches drives the search for new products and processes with potential pharmacological and for environmental bioremediation. This was evident by the ability of some isolates to inhibit three out of four investigated enteropathogenic species, besides *Xanthomonas* and *Fusarium* strains. Analysis of genomic composition and metabolites produced by these isolates could uncover new metabolic pathways associated with the synthesis of new biomolecules of pharmaceutical interest. In the same way, the isolates associated with production of IAA and siderophores may be used as bacterials consortia to induce native plant growth in areas degradeted by human action (de-Bashan et al., 2012).

## Conclusion

The integration of biological data found in this study suggests a hypothetical complex network of interaction and mutual dependence between the niches analyzed and the isolated bacteria. Classically, holoparasitic plants draw all necessary nutrients from their host plant. However, the results of this study show that the microbiota in *L. hypogaea* can also be of benefit by supplying nutrients essential for the survival of the plant. This hypothesis needs further and in-depth studies to become valid. From an ecological perspective, this is the first report of the potential of bacteria isolated from the IQ region in producing these siderophores and IAA, which allowed us to infer that part of the adaptive process of these plants in ferroginous fields can be a result of the ability of a large percentage of isolates to produce these compounds. Siderophores would be key for the chelation of iron in the Fe^3+^ state, existing in high concentrations in these ferroginous fields, and the production of IAA by a large number of isolates demonstrated how essential this compound would be for the induction of extensive root system of plants that survive in this environment. The characteristics of the soil from this area are extremely adverse, with low supply of water, requiring the plants to obtain nutrients from more superficial regions. Thus, it is possible that the survival of plants in this environment have some relationship with the presence and interaction with these microorganisms, which could also justify the large endemic plant found in this environment. From a biotechnological aspect, the perspectives and results obtained with this work point to the potential of developing a bacterial consortium that could be used as an indispensable tool in the recovery of areas degraded by anthropic actions, especially in this region where mining activities are eliminating important species of the biome of ferroginous fields. Therefore, the results presented in this work emphasize the importance of studying biological models neglected and differentiated such as the holoparasite plants and ferruginous soils from IQ since they are propitious sources for finding new compounds with biotechnological potential.

## Author Contributions

ÉF and LF collected samples. ÉF and LM conceived and designed the experiments. ÉF and LM performed all experiments and analysis. ÉF, LM, IS, and LR contributed with reagents, materials and analysis tools. ÉF and LM prepared the figures and tables. ÉF, LM, and SR wrote the paper.

## Conflict of Interest Statement

The authors declare that the research was conducted in the absence of any commercial or financial relationships that could be construed as a potential conflict of interest.

## Supplementary Material

FIGURE S1 **Geographical location of Serra da Brigida, collection site of *L. hypogaea***. Serra da Brigida is located around the city of Ouro Preto, state of Minas Gerais – Brazil. The stars indicate the sampling points for *Langsdorffia hypogaea*. Adaptated from Atlas Digital GeoAmbiental (http://institutopristino.org.br/atlas/).

FIGURE S2 **Presence of *nifH* confirmed by PCR analysis**.

## References

[B1] Altschul S. F. Madden T. L. Schaffer A. A. Zhang J. H. Zhang Z. Miller W. (1997). Gapped BLAST and PSI-BLAST: a new generation of protein database search programs. *Nucleic Acids Res.* 25 3389–3402. 10.1093/nar/25.17.3389 9254694 PMC146917

[B2] Anisimova M. Gascuel O. (2006). Approximate likelihood-ratio test for branches: a fast, accurate, and powerful alternative. *Syst. Biol.* 55 539–552. 10.1080/10635150600755453 16785212

[B3] Arbeli Z. Fuentes C. L. (2007). Improved purification and PCR amplification of DNA from environmental samples. *FEMS Microbiol. Lett.* 272 269–275. 10.1111/j.1574-6968.2007.00764.x 17521406

[B4] Badri D. V. Weir T. L. van der Lelie D. Vivanco J. M. (2009). Rhizosphere chemical dialogues: plant-microbe interactions. *Curr. Opin. Biotechnol.* 20 642–650. 10.1016/j.copbio.2009.09.014 19875278

[B5] Bais H. P. Weir T. L. Perry L. G. Gilroy S. Vivanco J. M. (2006). The role of root exudates in rhizosphere interactions with plants and other organisms. *Annu. Rev. Plant Biol.* 57 233–266. 10.1146/annurev.arplant.57.032905.105159 16669762

[B6] Bakker A. W. Schippers B. (1987). Microbial cyanide production in the rhizosphere in relation to potato yield reduction and *Pseudomonas* spp-mediated plant growth-stimulation. *Soil Biol. Biochem.* 19 451–457. 10.1016/0038-0717(87)90037-X

[B7] Bashan Y. de-Bashan L. E. (2005). “Bacteria,” in *Encyclopedia of Soils in the Environment*, ed. Hillel D. (Oxford: Elsevier), 103–115.

[B8] Bashan Y. Holguin G. (1998). Proposal for the division of plant growth-promoting rhizobacteria into two classifications: biocontrol-PGPB (plant growth-promoting bacteria) and PGPB. *Soil Biol. Biochem.* 30 1225–1228. 10.1016/S0038-0717(97)00187-9

[B9] Batmanian G. J. Haridasan M. (1985). Primary production and accumulation of nutrients by the ground layer community of cerrado vegetation of central Brazil. *Plant Soil* 88 437–440. 10.1007/Bf02197500

[B10] Bertin C. Yang X. H. Weston L. A. (2003). The role of root exudates and allelochemicals in the rhizosphere. *Plant Soil* 256 67–83. 10.1023/A:1026290508166

[B11] Bhardwaj D. Ansari M. W. Sahoo R. K. Tuteja N. (2014). Biofertilizers function as key player in sustainable agriculture by improving soil fertility, plant tolerance and crop productivity. *Microb. Cell Fact.* 13:66 10.1186/1475-2859-13-66 PMC4022417 24885352

[B12] Bhattacharyya P. N. Jha D. K. (2012). Plant growth-promoting rhizobacteria (PGPR): emergence in agriculture. *World J. Microbiol. Biotechnol.* 28 1327–1350. 10.1007/s11274-011-0979-9 22805914

[B13] Bric J. M. Bostock R. M. Silverstone S. E. (1991). Rapid in situ assay for indoleacetic acid production by bacteria immobilized on a nitrocellulose membrane. *Appl. Environ. Microbiol.* 57 535–538. 16348419 10.1128/aem.57.2.535-538.1991 PMC182744

[B14] Burgmann H. Widmer F. Von Sigler W. Zeyer J. (2004). New molecular screening tools for analysis of free-living diazotrophs in soil. *Appl. Environ. Microbiol.* 70 240–247. 10.1128/AEM.70.1.240-247.2004 14711647 PMC321232

[B15] Cardoso L. J. T. (2014). *Balanophoraceae no Brasil.* Master thesis, Jardim Botânico do Rio de Janeiro, Rio de Janeiro.

[B16] Cardoso L. J. T. Alves R. J. V. Braga J. M. A. (2011). A new species and a key for *Langsdorffia* (Balanophoraceae). *Syst. Bot.* 36 424–427. 10.1600/036364411X569606

[B17] Castresana J. (2000). Selection of conserved blocks from multiple alignments for their use in phylogenetic analysis. *Mol. Biol. Evol.* 17 540–552. 10.1093/oxfordjournals.molbev.a026334 10742046

[B18] Cespedes C. L. Alarcon J. Aqueveque P. M. Lobo T. Becerra J. Balbontin C. (2015). New environmentally-friendly antimicrobials and biocides from Andean and Mexican biodiversity. *Environ. Res.* 142 ER15301. 10.1016/j.envres.2015.08.004 26298556

[B19] Chevenet F. Brun C. Banuls A. L. Jacq B. Christen R. (2006). TreeDyn: towards dynamic graphics and annotations for analyses of trees. *BMC Bioinformatics* 7:439 10.1186/1471-2105-7-439 PMC1615880 17032440

[B20] Choudhary D. K. Prakash A. Johri B. N. (2007). Induced systemic resistance (ISR) in plants: mechanism of action. *Indian J. Microbiol.* 47 289–297. 10.1007/s12088-007-0054-2 23100680 PMC3450033

[B21] Cigna J. Raoul des Essarts Y. Mondy S. Helias V. Beury-Cirou A. Faure D. (2015). Draft Genome sequences of *Pseudomonas fluorescens* strains PA4C2 and PA3G8 and *Pseudomonas putida* PA14H7, three biocontrol bacteria against *Dickeya* phytopathogens. *Genome Announc.* 3:e01503-14. 10.1128/genomeA.01503-14 PMC4319517 25635023

[B22] Compant S. Duffy B. Nowak J. Clement C. Barka E. A. (2005). Use of plant growth-promoting bacteria for biocontrol of plant diseases: principles, mechanisms of action, and future prospects. *Appl. Environ. Microbiol.* 71 4951–4959. 10.1128/Aem.71.9.4951-4959.2005 16151072 PMC1214602

[B23] Costacurta A. Vanderleyden J. (1995). Synthesis of phytohormones by plant-associated bacteria. *Crit. Rev. Microbiol.* 21 1–18. 10.3109/10408419509113531 7576148

[B24] Crowley D. E. Wang Y. C. Reid C. P. P. Szaniszlo P. J. (1991). Mechanisms of iron acquisition from siderophores by microorganisms and plants. *Plant Soil* 130 179–198. 10.1007/BF00011873

[B25] Dalmaso G. Z. L. Ferreira D. Vermelho A. B. (2015). Marine extremophiles: a source of hydrolases for biotechnological applications. *Mar. Drugs* 13 1925–1965. 10.3390/md13041925 25854643 PMC4413194

[B26] de-Bashan L. E. Hernandez J. P. Bashan Y. (2012). The potential contribution of plant growth-promoting bacteria to reduce environmental degradation – A comprehensive evaluation. *Appl. Soil Ecol.* 61 171–189. 10.1016/j.apsoil.2011.09.003

[B27] Dobereiner J. Marriel I. E. Nery M. (1976). Ecological distribution of *Spirillum*-Lipoferum Beijerinck. *Can. J. Microbiol.* 22 1464–1473. 10.1139/m76-217 10062

[B28] Dodd I. C. Ruiz-Lozano J. M. (2012). Microbial enhancement of crop resource use efficiency. *Curr. Opin. Biotechnol.* 23 236–242. 10.1016/j.copbio.2011.09.005 21982722

[B29] Doyle J. Doyle J. (1987). A rapid procedure for DNA purification from small quantities of fresh leaf tissue. *Phytochem. Bull.* 19 11–15.

[B30] Edgar R. C. (2004). MUSCLE: multiple sequence alignment with high accuracy and high throughput. *Nucleic Acids Res.* 32 1792–1797. 10.1093/nar/gkh340 15034147 PMC390337

[B31] Ewing B. Green P. (1998). Base-calling of automated sequencer traces using phred. II. Error probabilities. *Genome Res.* 8 186–194. 10.1101/gr.8.3.186 9521922

[B32] Ewing B. Hillier L. Wendl M. C. Green P. (1998). Base-calling of automated sequencer traces using phred. I. Accuracy assessment. *Genome Res.* 8 175–185. 10.1101/gr.8.3.175 9521921

[B33] Ferreira M. T. M. (2011). *Composição Florística e Distribuição Vertical de Epífitas Vasculares Sobre Indivíduos de Guapira opposita (vell.) Reitz (Nyctaginaceae) em um Fragmento Florestal na Serra da Brígida, Ouro Preto, MG*. Master thesis, Universidade Federal de Ouro Preto, Ouro Preto.

[B34] Filho A. C. Curi N. Shinzato E. (2010). Soil landscape relationships at the Quadrilátero Ferrífero in the state of Minas Gerais, Brazil. *Pesqui. Agropecu. Bras.* 45 903–916.

[B35] Fine P. V. Mesones I. Coley P. D. (2004). Herbivores promote habitat specialization by trees in Amazonian forests. *Science* 305 663–665. 10.1126/science.1098982 15286371

[B36] George P. Gupta A. Gopal M. Thomas L. Thomas G. V. (2013). Multifarious beneficial traits and plant growth promoting potential of *Serratia marcescens* KiSII and *Enterobacter* sp. *RNF* 267 isolated from the rhizosphere of coconut palms (*Cocos nucifera* L.). *World J. Microbiol. Biotechnol.* 29 109–117. 10.1007/s11274-012-1163-6 22948479

[B37] Glick B. R. (2010). Using soil bacteria to facilitate phytoremediation. *Biotechnol. Adv.* 28 367–374. 10.1016/j.biotechadv.2010.02.001 20149857

[B38] Gonçalves J. A. C. Lena J. C. (2013). Evaluation of risk to human health by natural arsenic contamination in groundwater and soils from the urban area of Ouro Preto (MG). *Geol. USP. Série Cient.* 13 145–158.

[B39] Goodland R. (1971). A physiognomic analysis of the ‘Cerrado’ vegetation of central Brasil. *J. Ecol.* 59 411–419. 10.2307/2258321

[B40] Goodland R. Pollard R. (1973). The Brazilian Cerrado vegetation: a fertility gradient. *J. Ecol.* 61 219–224. 10.1590/S1519-69842013000200007

[B41] Greenslade P. J. M. (1983). Adversity selection and the habitat templet. *Am. Nat.* 122 352–365. 10.1086/284140

[B42] Hansen B. (1980). Balanophoraceae. *Flora Neotrop. Monogr.* 23:80.

[B43] Haridasan M. (2008). Nutritional adaptations of native plants of the cerrado biome in acid soils. *Braz. J. Plant Physiol.* 20 183–195. 10.1590/S1677-04202008000300003

[B44] Herms D. A. Mattson W. J. (1992). The dilemma of plants - to grow or defend. *Q. Rev. Biol.* 67 478–478. 10.1086/417659

[B45] Hibbing M. E. Fuqua C. Parsek M. R. Peterson S. B. (2010). Bacterial competition: surviving and thriving in the microbial jungle. *Nat. Rev. Microbiol.* 8 15–25. 10.1038/nrmicro2259 19946288 PMC2879262

[B46] Hsiao S. C. Mauseth J. D. Peng C. I. (1995). Composite bundles, the host-parasite interface in the holoparasitic angiosperms *Langsdorffia* and *Balanophora* (Balanophoraceae). *Am. J. Bot.* 82 81–91. 10.2307/2445790

[B47] Jacobi C. M. do Carmo F. F. (2008). Diversidade dos campos rupestres ferruginosos no Quadrilátero Ferrífero, MG. *Megadiversidade* 4 25–33.

[B48] Ji S. H. Gururani M. A. Chun S. C. (2014). Isolation and characterization of plant growth promoting endophytic diazotrophic bacteria from Korean rice cultivars. *Microbiol. Res.* 169 83–98. 10.1016/j.micres.2013.06.003 23871145

[B49] Jones D. L. Edwards A. C. Donachie K. Darrah P. R. (1994). Role of proteinaceous amino-acids released in root exudates in nutrient acquisition from the rhizosphere. *Plant Soil* 158 183–192. 10.1007/BF00009493

[B50] Kim K. Jang Y. J. Lee S. M. Oh B. T. Chae J. C. Lee K. J. (2014). Alleviation of salt stress by *Enterobacter* sp. EJ01 in tomato and *Arabidopsis* is accompanied by up-regulation of conserved salinity responsive factors in plants. *Mol. Cells* 37 109–117. 10.14348/molcells.2014.2239 24598995 PMC3935623

[B51] Kloepper J. W. Leong J. Teintze M. Schroth M. N. (1980). Enhanced plant-growth by siderophores produced by plant growth-promoting rhizobacteria. *Nature* 286 885–886. 10.1038/286885a0

[B52] Korfiatis K. J. Stamou G. P. (1999). Habitat templets and the changing worldview of ecology. *Biol. Philos.* 14 375–393. 10.1023/A:1006543127454

[B53] Lane D. J. Pace B. Olsen G. J. Stahl D. A. Sogin M. L. Pace N. R. (1985). Rapid-determination of 16s ribosomal-Rna sequences for phylogenetic analyses. *Proc. Natl. Acad. Sci. U.S.A.* 82 6955–6959. 10.1073/pnas.82.20.6955 2413450 PMC391288

[B54] Lodewyckx C. Vangronsveld J. Porteous F. Moore E. R. B. Taghavi S. Mezgeay M. (2002). Endophytic bacteria and their potential applications. *Crit. Rev. Plant Sci.* 21 583–606. 10.1080/0735-260291044377

[B55] Lopez Pascua L. Hall A. R. Best A. Morgan A. D. Boots M. Buckling A. (2014). Higher resources decrease fluctuating selection during host-parasite coevolution. *Ecol. Lett.* 17 1380–1388. 10.1111/ele.12337 25167763 PMC4257576

[B56] Lynch J. M. Whipps J. M. (1990). Substrate flow in the rhizosphere. *Plant Soil* 129 1–10. 10.1007/Bf00011685

[B57] Magnus V. Simaga S. Iskric S. Kveder S. (1982). Metabolism of tryptophan, indole-3-acetic acid, and related compounds in parasitic plants from the genus *Orobanche*. *Plant Physiol.* 69 853–858. 10.1104/pp.69.4.853 16662308 PMC426317

[B58] Maniatis T. Fritsch E. F. Sambrook J. (1982). *Molecular Cloning: A Laboratory Manual*. New York, NY: Cold Spring Harbor Laboratory Press.

[B59] Meldau D. G. Long H. H. Baldwin I. T. (2012). A native plant growth promoting bacterium, *Bacillus* sp. B55, rescues growth performance of an ethylene-insensitive plant genotype in nature. *Front. Plant Sci.* 3:112 10.3389/fpls.2012.00112 PMC3371617 22701461

[B60] Miles L. Grainger A. Phillips O. (2004). The impact of global climate change on tropical forest biodiversity in Amazonia. *Glob. Ecol. Biogeogr.* 13 553–565. 10.1111/gcb.13315

[B61] Mirza M. S. Ahmad W. Latif F. Haurat J. Bally R. Normand P. (2001). Isolation, partial characterization, and the effect of plant growth-promoting bacteria (PGPB) on micro-propagated sugarcane in vitro. *Plant Soil* 237 47–54. 10.1023/A:1013388619231

[B62] Moore J. C. McCann K. Setala H. De Ruiter P. C. (2003). Top-down is bottom-up: does predation in the rhizosphere regulate aboveground dynamics? *Ecology* 84 846–857. 10.1890/0012-96582003084[0846:TIBDPI]2.0.CO;2

[B63] Nadeem S. M. Ahmad M. Zahir Z. A. Javaid A. Ashraf M. (2014). The role of mycorrhizae and plant growth promoting rhizobacteria (PGPR) in improving crop productivity under stressful environments. *Biotechnol. Adv.* 32 429–448. 10.1016/j.biotechadv.2013.12.005 24380797

[B64] Neilands J. B. (1995). Siderophores – Structure and function of microbial iron transport compounds. *J. Biol. Chem.* 270 26723–26726. 10.1074/jbc.270.45.26723 7592901

[B65] Nickrent D. L. (2002). “Plantas parásitas en el mundo,” in *Plantas Parásitas de la Península Ibérica e Islas Baleares*, eds López-Sáez J. A. Catalán P. Sáez L. (Madrid: Mundi-Prensa Libros), 7–28.

[B66] Perez-Montano F. Alias-Villegas C. Bellogin R. A. del Cerro P. Espuny M. R. Jimenez-Guerrero I. (2014). Plant growth promotion in cereal and leguminous agricultural important plants: from microorganism capacities to crop production. *Microbiol. Res.* 169 325–336. 10.1016/j.micres.2013.09.011 24144612

[B67] Pianka E. R. (1970). On r and K selection. *Am. Nat.* 104 592–597. 10.1086/282697

[B68] Pierre J. L. Baret P. Serratrice G. (2003). Hydroxyquinolines as iron chelators. *Curr. Med. Chem.* 10 1077–1084. 10.2174/0929867033457584 12678678

[B69] Pott A. Pott V. J. Sobrinho A. A. B. (2004). “Plantas úteis à sobrevivência no Pantanal,” in *Proceedings of the IV Simpósio Sobre Recursos Naturais e Sócio-Econômicos do Pantanal*, Corumbá, 39–40.

[B70] Raaijmakers J. M. Paulitz T. C. Steinberg C. Alabouvette C. Moenne-Loccoz Y. (2009). The rhizosphere: a playground and battlefield for soilborne pathogens and beneficial microorganisms. *Plant Soil* 321 341–361. 10.1007/s11104-008-9568-6

[B71] Ramamoorthy V. Viswanathan R. Raguchander T. Prakasam V. Samiyappan R. (2001). Induction of systemic resistance by plant growth promoting rhizobacteria in crop plants against pests and diseases. *Crop Prot.* 20 1–11. 10.1016/S0261-2194(00)00056-9

[B72] Ribeiro S. P. Brown V. K. (2006). Prevalence of monodominant vigorous tree populations in the tropics: herbivory pressure on *Tabebuia* species in very different habitats. *J. Ecol.* 94 932–941. 10.1111/j.1365-2745.2006.01133.x

[B73] Richardson A. E. Barea J. M. McNeill A. M. Prigent-Combaret C. (2009). Acquisition of phosphorus and nitrogen in the rhizosphere and plant growth promotion by microorganisms. *Plant Soil* 321 305–339. 10.1007/s11104-009-9895-2

[B74] Rodriguez H. Fraga R. (1999). Phosphate solubilizing bacteria and their role in plant growth promotion. *Biotechnol. Adv.* 17 319–339. 10.1016/S0734-9750(99)00014-2 14538133

[B75] Rosière C. A. Chemale F. Jr. (2000). Itabiritos e minérios de ferro de alto teor do Quadrilátero ferrífero – uma visão geral e discussão. *Geonomos* 8 27–43.

[B76] Saleem M. Arshad M. Hussain S. Bhatti A. S. (2007). Perspective of plant growth promoting rhizobacteria (PGPR) containing ACC deaminase in stress agriculture. *J. Ind. Microbiol. Biotechnol.* 34 635–648. 10.1007/s10295-007-0240-6 17665234

[B77] Schwyn B. Neilands J. B. (1987). Universal chemical-assay for the detection and determination of siderophores. *Anal. Biochem.* 160 47–56. 10.1016/0003-2697(87)90612-9 2952030

[B78] Sema/Gtz. (1995). *Lista Vermelha de Plantas Ameaçadas de Extinção no Estado do Paraná*. Curitíba: SEMA/GTZ.

[B79] Southwood T. R. E. (1977). Habitat, the template for ecological strategies? *J. Anim. Ecol.* 46 337–365. 10.2307/3817

[B80] Strauss M. L. A. Jolly N. P. Lambrechts M. G. van Rensburg P. (2001). Screening for the production of extracellular hydrolytic enzymes by non-*Saccharomyces* wine yeasts. *J. Appl. Microbiol.* 91 182–190. 10.1046/j.1365-2672.2001.01379.x 11442729

[B81] Sukweenadhi J. Kim Y. J. Choi E. S. Koh S. C. Lee S. W. Yang D. C. (2015). *Paenibacillus* yonginensis DCY84(T) induces changes in *Arabidopsis thaliana* gene expression against aluminum, drought, and salt stress. *Microbiol. Res.* 172 7–15. 10.1016/j.micres.2015.01.007 25721473

[B82] Taylor D. R. Aarssen L. W. Loehle C. (1990). On the relationship between R/K selection and environmental carrying-capacity – a new habitat templet for plant life-history strategies. *Oikos* 58 239–250. 10.2307/3545432

[B83] Teather R. M. Wood P. J. (1982). Use of congo red polysaccharide interactions in enumeration and characterization of cellulolytic bacteria from the bovine rumen. *Appl. Environ. Microbiol.* 43 777–780. 7081984 10.1128/aem.43.4.777-780.1982 PMC241917

[B84] Vacheron J. Desbrosses G. Bouffaud M. L. Touraine B. Moenne-Loccoz Y. Muller D. (2013). Plant growth-promoting rhizobacteria and root system functioning. *Front. Plant Sci.* 4:356 10.3389/fpls.2013.00356 PMC3775148 24062756

[B85] Vale P. N. C. (2013). *Solo e Topografia Como Condicionantes da Distribuição da Vegetação em Fitofisionomias Campestre e Florestal em Contato Direto na Serra da Brígida, Ouro Preto, MG*. Master, thesis, Universidade Federal de Ouro Preto, Ouro Preto.

[B86] Verdouw H. Vanechteld C. J. A. Dekkers E. M. J. (1978). Ammonia determination based on indophenol formation with sodium salicylate. *Water Res.* 12 399–402. 10.1016/0043-1354(78)90107-0

[B87] Vessey J. K. (2003). Plant growth promoting rhizobacteria as biofertilizers. *Plant Soil* 255 571–586. 10.1023/A:1026037216893

[B88] Vince A. Dawson A. M. Park N. O’Grady F. (1973). Ammonia production by intestinal bacteria. *Gut* 14 171–177. 10.1136/gut.14.3.171 4573343 PMC1412620

[B89] Voisard C. Keel C. Haas D. Defago G. (1989). Cyanide production by *Pseudomonas fluorescens* helps suppress black root rot of tobacco under gnotobiotic conditions. *EMBO J.* 8 351–358. 16453871 10.1002/j.1460-2075.1989.tb03384.x PMC400813

[B90] Wang S. Ouyang L. Ju X. Zhang L. Zhang Q. Li Y. (2014). Survey of plant drought-resistance promoting bacteria from *Populus euphratica* tree living in arid area. *Indian J. Microbiol.* 54 419–426. 10.1007/s12088-014-0479-3 25320440 PMC4186926

[B91] Zhang Y. Ruyter-Spira C. Bouwmeester H. J. (2014). Engineering the plant rhizosphere. *Curr. Opin. Biotechnol.* 32C, 136–142. 10.1016/j.copbio.2014.12.006 25555138

[B92] Zhao Y. Selvaraj J. N. Xing F. Zhou L. Wang Y. Song H. (2014). Antagonistic action of *Bacillus subtilis* strain SG6 on *Fusarium graminearum*. *PLoS ONE* 9:e92486 10.1371/journal.pone.0092486 PMC3961383 24651513

[B93] Zilber-Rosenberg I. Rosenberg E. (2008). Role of microorganisms in the evolution of animals and plants: the hologenome theory of evolution. *FEMS Microbiol. Rev.* 32 723–735. 10.1111/j.1574-6976.2008.00123.x 18549407

