# Supplementary figures and images for: Plant Growth Promoting Bacteria Associated with Langsdorffia hypogaea-Rhizosphere-Host Biological Interface: A Neglected Model of Bacterial Prospection
Source: Front Microbiol. 2017 Feb 10;8:172. doi: 10.3389/fmicb.2017.00172 (PMC5300976; doi:10.3389/fmicb.2017.00172)

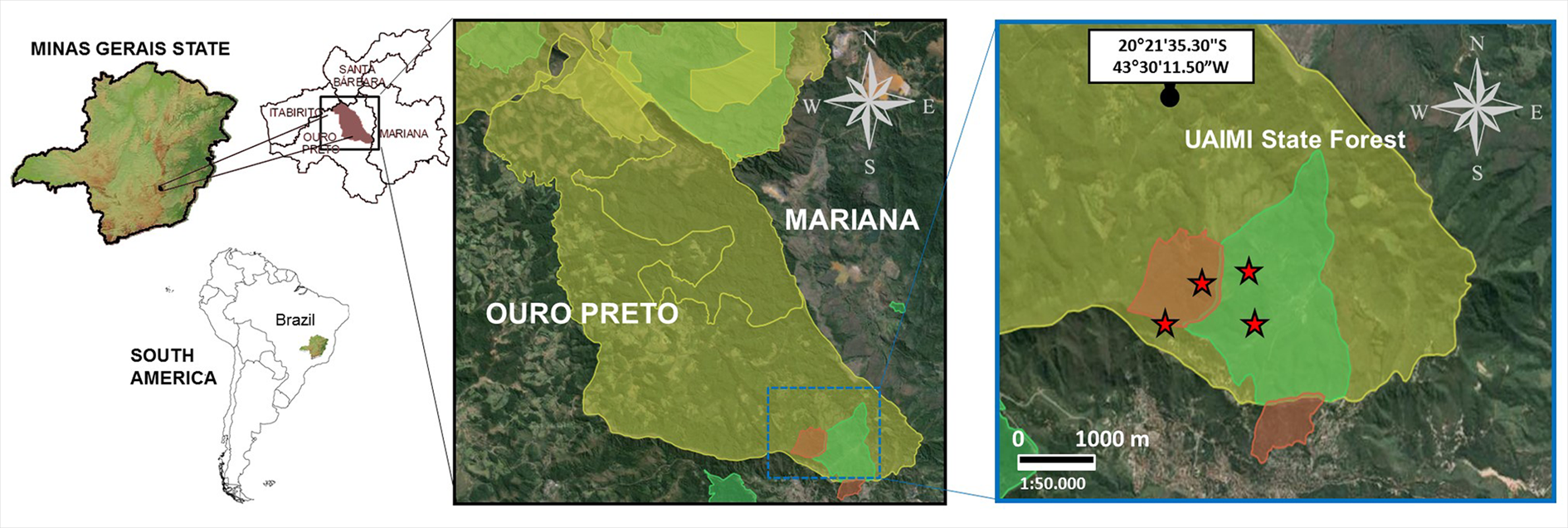

Supplement: FIGURE S1 — Geographical location of Serra da Brigida, collection site of L. hypogaea. Serra da Brigida is located around the city of Ouro Preto, state of Minas Gerais – Brazil. The stars indicate the sampling points for Langsdorffia hypogaea. Adaptated from Atlas Digital GeoAmbiental (http://institutopristino.org.br/atlas/). [file Image_1.TIF]

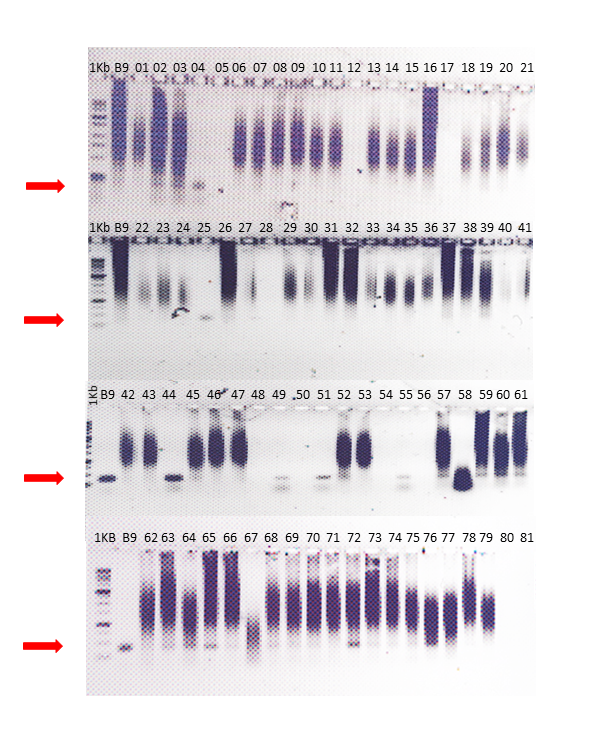

Supplement: FIGURE S2 — Presence of nifH confirmed by PCR analysis. [file Image_2.TIF]
